# To be or not to be: relationship between grandparent status and health and wellbeing

**DOI:** 10.1186/s12877-021-02052-w

**Published:** 2021-03-24

**Authors:** Daniel W. L. Lai, Jia Li, Xue Bai

**Affiliations:** 1 Faculty of Social Sciences, Hong Kong Baptist University, Kowloon, Hong Kong; 2 The Jockey Club School of Public Health and Primary Car, The Chinese University of Hong Kong, New Territories, Hong Kong; 3 Department of Applied Social Sciences, The Hong Kong Polytechnic University, 11 Yuk. Choi Road, Hung Hum, Kowloon, Hong Kong

**Keywords:** Grandparenthood, Resilience, Happiness, Self-rated physical health, Hong Kong Chinese, Telephone survey

## Abstract

**Background:**

It is common for older people to become grandparents in later life. However, the impacts of grandparenting on their health and well-being remain ambiguous, especially in Chinese society, where the family is in the core of culture. The current study explored the relationship between grandparenthood and Chinese older people’s health and psychological well-being in Hong Kong.

**Methods:**

Cross-sectional data were collected from a sample of 1208 Hong Kong Chinese older people aged 55 and above through a telephone survey conducted in 2019. Participants were grouped into three categories: current grandparents (*n* = 507), grandparents-to-be (*n* = 275), and grandparents-not-to-be (*n* = 426). Multivariate linear regressions were performed to examine the relationship between grandparenting status and health and well-being outcomes, including self-rated physical health, mental health, resilience, and happiness. The potential moderating roles of older adults’ demographic characteristics, including age, sex, education, marital status, financial status, were also examined.

**Results:**

Bivariate analyses suggested statistically significant differences between health and well-being across the three groups of participants. Regression models showed that, compared with grandparents-not-to-be, being a current grandparent was associated with a significantly higher happiness level. Being a future grandparent was associated with significantly higher levels of happiness, resilience, and self-rated physical health. Moderating analyses showed that age, marital status, and educational level could moderate the relationship between grandparent status and resilience and self-rated mental health.

**Conclusions:**

The current study offers preliminary insights into the significant relationship between grandparenthood and older adults’ health and well-being. It calls for future studies to further explore the mechanisms between grandparenthood and the healthy ageing of different subgroups of older adults.

**Supplementary Information:**

The online version contains supplementary material available at 10.1186/s12877-021-02052-w.

## Background

In view of current population ageing in many countries, previous research has shown that the majority of people aged 50 and older are facing or have faced the transition to grandparenthood, which plays a crucial role in later life [1–3]. It is not uncommon for grandparents to become caregivers for their grandchildren, especially in societies with a family-oriented culture, such as in Chinese societies [4]. There has been some attention to how taking up caring roles as grandparents affects older adults’ well-being and health [1, 5]. However, the impacts of grandparenting on older adults themselves remain inconclusive. This study attempts to contribute to a more detailed understanding of the impacts of grandparenting involvement on older adults’ well-being in Hong Kong.

Existing literature has documented the benefits of grandparenting on older adults, such as positive levels of physical activity and self-care [6], caregiver satisfaction [7], and improved health habits [8]. Timberlake (1981) summarized eight benefits of grandparenthood, including but not limited to the provision of stability, a goal, excitement, sense of achievement, and an opportunity to help to one’s life [9]. According to the role enhancement theory, being a grandparent can bring out an older adult purpose of life and a sense of meaning [10]. The needs of generativity can also bring older adults a sense of satisfaction when they become grandparents [11]. In addition, children and grandchildren can also serve as critical emotional and social sources for older adults [12–14]. Empirically, researchers have found positive correlations between intergenerational support and subjective well-being [15, 16] and mental health [17, 18] among older adults. For instance, Lai, Lee, Li, and Dong [19] found that a stronger sense of closeness with grandchildren was associated with self-rated health status and quality of life among older Chinese immigrants in the United States. In South Korea, researchers have found that grandparents caring for grandchildren tended to perceive meaning in their lives and reported lower levels of stress and depressive mood [20]. A German study by Mahne and Huxold [13] reported that contact frequency and emotional closeness with grandchildren boosted positive aspects of subjective well-being. A qualitative study in Zimbabwe found that grandparents attained various protective factors for their everyday health and wellbeing, such as personal resources, resilient coping strategies, and spirituality [14].

Additionally, some studies have drawn attention to the potential adverse effects of becoming a grandparent. For instance, in line with the role strain theory, a study in Thailand has found that providing regular care to a grandchild is related to lower self-rated health and more functional limitation [21]). Grandparents who perceived and experienced high caregiving-related stress can experience emotional distress [22, 23]. The sudden transition to grandparenthood can result in role confusion and adversely impact mental health [22, 24]. A high level grandparenting involvement may also trigger intergenerational conflict with adult children. It is more commonly found in Chinese culture wherein grandparents tend to discipline the grandchild [25].

In addition, becoming a grandparent can have mixed effects on different groups of older adults’ well-being. For example, some research has shown that the transition to first-time grandparenthood positively affects well-being and depressive symptoms among women but not men [1, 26]. Grandparents with lower education and worse financial status may suffer from more negative effects of grandparenting [21]. Sheppard and Monden [26] found a positive association between becoming a grandparent and an increased subjective life expectancy for men, but only for those who were employed. A Chinese study has found that urban grandfathers are more likely to enjoy the health benefits of grandparenting, and rural grandmothers suffer health risks from intergenerational caregiving [27]. Another study has also confirmed the protective effect of living in urban areas [28]. In summary, the impacts of grandparenting on older adults are not universal across subgroups, and moderators in the grandparenting-health relationship need to be further examined.

Not many studies have been conducted in Hong Kong regarding the impacts of grandparenting on older adults’ health and well-being previously. As exceptions, a qualitative study conducted among Hong Kong grandparents has revealed both enjoyment and stress reported by the participants. For those who need to take an active role in caregiving, grandparenting is demanding physically, socially, and psychologically [29]. A quantitative study has found that grandparenting rewards can be protective for older adults against late-life depression [30].

Even though Chinese account for the vast majority of Hong Kong society, due to the low birth rate, small housing size, and the prevalence of foreign domestic helpers, grandparents are more likely to be the secondary instead of the primary carer [31]. According to a report in 2017, 32% of Hong Kong parents agreed to let their parents help raise their children, while 48% disagreed. From 2013 to 2017, the agreement that Hong Kong parents appreciated grandparents’ help dropped by 19%. Only 29% of the parents agree that grandparents should be closely involved in how the children would be brought up [32]. While co-living and custodial grandparents are also less common in Hong Kong than in Mainland China, but the needs for grandparents’ involvement in child care are growing in Hong Kong due to the increasing pressure regarding child care [33].

In summary of previous studies, analyses of whether and how grandparenthood is associated with older people’s well-being and health have yielded inconsistent results. Previous studies call for more attention to how various factors, such as individuals’ socio-economic status, influence the effects of grandparenting on older adults [1, 26]. This study aimed to provide further evidence with data from Hong Kong. The objective of this study includes (1) to understand the relationship between grandparenthood and older people’s health and well-being; (2) to investigate the factors that can moderate the relationship between grandparenthood and older adults’ health and well-being.

## Methods

### Research design, target population, and sampling

This study used a cross-sectional quantitative survey design, with data collected through telephone surveys from June to August 2019. The target population consisted of older people aged 55 and older who were also legal citizens residing in Hong Kong at the time of the survey and able to communicate in Cantonese. Randomly generated telephone numbers were identified. These numbers were generated based upon the know prefixes as included in the Numbering Plan of the Office of the Communications Authority in Hong Kong. All telephone numbers obtained from the Numbering Plan were pre-screened to detect the tritone signal for invalid numbers. Computer screening results were merged with a previously obtained database of actual manual dialing records to eliminate invalid numbers. Known business lines gathered from previous dialing records or publicly available sources were also excluded. Finally, the estimate required amount of telephone numbers were randomly drawn from the database to form the final sampling frame.

For second-level sampling, when telephone contact was successfully established with a target household, one qualified respondent was selected using the ‘next birthday rule’ to further eliminate potential bias. To ensure the representativeness of the final sample, the raw data collected were rim-weighted according to the latest sex-age distribution of the Hong Kong population aged 55 or older and educational attainment distributions [34]. The sample size calculation was based on the number of Hong Kong Chinese population older than 55, which was 246, 0300 by the end of 2018 [35]. A total of 1067 participants was needed to ensure the sampling error of the percentages obtained would be within no more than +/− 3% based on the full sample at a 95% confidence level.

### Data collection

An anonymous structured questionnaire was used for data collection (please refer to Additional file 1). The data collection was entrusted to a professional survey firm that made use of telephone interviews in this study. About 50 questions were used in the questionnaire, both the content was consulted about half a dozen of older adults fall into the inclusion criteria of the study. To complete the questionnaire, about 15 min was required. The professional survey firm would made up to five attempts to call a number a different times within a week before considered the number was dropped as unable to be contacted. The standard protocol of the professional survey firm would adopt various monitoring methods to ensure quality of interviewers’ performance during data collection process, and they included live supervision, audio and video recording, and montoring of data collection screen activities. Ethical approval was obtained from the Human Subjects Ethics Subcommittee of the authors’ home university. Due to the telephone interview nature of the data collection, verbal consent instead of written consent was used at the beginning of the data collection.

### Measures

Grandparent status was determined by asking the participants to self-identify as a current grandparent (having at least one grandchild), future grandparent-to-be (answering “*yes*” to the question “*do you expect to be a grandparent in the future*?”), or not expected to be a grandparent. Health and wellbeing variables included self-rated physical health, self-rated mental health, happiness, and resilience. Self-rated physical health was measured by a single item asking participants to rate their physical health along a 5-point scale (1 = very poor, 5 = very good), with a higher score indicating better physical health. Self-rated mental health was measured by a single item asking participants to rate their mental health along a 5-point scale (1 = very poor, 5 = very good), with a higher score indicating better mental health. Single-item self-rated health status measures have been found highly correlated with objective measures and are reliable instruments [36, 37]. Happiness was measured using the Chinese version of the Subjective Happiness Scale [38], consisting of four items, each rated along a 7-point scale, with a higher score indicating a higher level of happiness. The scores for all items were summed to form an overall score ranging from 4 to 28. The Cronbach’s Alpha of the scale in this study was 0.798, indicating a good internal consistency. Resilience was measured by the Chinese version of the Connor-Davidson Resilience Scale [39], a two-item scale with a score range of 0 to 4 for each item. Total scores range from 0 to 8 with a higher score indicating a higher level of resilience. The Cronbach’s Alpha of the scale in this study was 0.673, indicating an acceptable internal consistency.

Based upon previous research results concerning predictors of health and wellbeing among older people, the effects a few confounding variables were also tested. These included sex, age, marital status, education level, self-rated financial status, and level of physical activity. Sex was grouped as either male or female. Age referred to participants’ chronological age, while marital status was grouped as either married or non-married (single/widowed/divorced). Education level was grouped into eight levels ranging from no formal education (1) to post-graduate level or above (8). Educational status was further re-categorized into three groups: lower than middle school, middle school, and higher than middle school. Self-rated financial status was measured by a single item asking participants to rate their level of financial adequacy along a 5-point scale (1 = very inadequate, 5 = very adequate). Level of physical activity was measured by asking participant to rate their general level of physical activity, including going out walking, house chores, and exercises along a 5-point scale ranging from very low (1) to very high (5).

### Data analysis

Single imputation method was adopted to fill in the missing value as the missing rate was minimal. Multiple imputation with five imputed datasets generated by Markov chain Monte Carlo method was also performed for sensitivity analysis. The results of the two methods were similar. Descriptive analysis was conducted to examine the demographic background of participants. Oneway analysis of variance tests were performed to compare differences between the three groups of participants. If the oneway tests were significant, Tukey’s HSD tests were further run to test the differences between each two groups. Hierarchal regression analysis was conducted for each of the well-being variables as the dependent variable. Independent variables include sex, age, education, financial status, marriage, physical activity, and grandparent status. The controlling variables were first entered into the model, followed by the grandparent status. 95% confidence intervals were calculated to estimate the magnitude of unstandardized coefficients. The increases in R^2^ of the regression models were examined. Interaction terms between grandparent status and five demographic characteristics, including age, sex, marital status, education, and finance were examined in the regression models, respectively, as well.

## Results

### Sample characteristics

Participants’ demographic characteristics are shown in Table 1. Current grandparents reported a significantly older age than the other two groups. Most participants (49.67%) had a middle school educational level. On a scale of 1 to 5, the average score for financial status was 3.31 (*standard deviation* = 0.75) and the average score for physical activity was 3.31 (standard deviation = 0.85). There were significant differences between the three groups in terms of age, education, marital status, and sex.

**Table 1:** Sample characteristics (*N* = 1208)

Variable	Overall (*N* = 1208)	Current (*n* = 507)	To-be (*n* = 275)	Not-to-be (*n* = 426)	Chisquare/ F-value
Age	66.58 (5.90)	68.77(5.90)	64.45(5.30)	65.36(5.49)	69.54^***^
Education					
< Middle school	303 (25.08%)	180 (35.50%)	43 (15.64%)	80 (18.78%)	64.204^***^
Middle school	600 (49.67%)	239 (47.14%)	154 (56.00%)	207 (48.59%)	
> Middle school	305 (25.25%)	88 (17.36%)	78 (28.36%)	139 (32.63%)	
Sex					
Female	628 (51.99%)	286 (56.41%)	116 (42.18%)	226 (53.05%)	14.76^***^
Male	580 (48.01%)	221 (43.59%)	159 (57.82%)	200 (46.95%)	
Marriage					
Married	1005 (83.20%)	444 (87.57%)	263 (95.64%)	298 (69.95%)	90.831^***^
Not married	203 (16.80%)	63 (12.43%)	12 (4.36%)	128 (30.05%)	
Financial status	3.31 ± 0.75	3.32 ± 0.73	3.38 ± 0.72	3.26 ± 0.80	2.46 ^ns^
Physical activity	3.31 ± 0.85	3.34 ± 0.87	3.33 ± 0.80	3.25 ± 0.84	1.45 ^ns^

Note: ^*^p < 0.05; ^**^p < 0.01; ^***^p < 0.001; *ns* not significant

### Differences between groups by grandparent status

According to oneway analsysis of variance tests (shown in Table 2), there are significant differences in levels of happiness (*p* < 0.001) between the three groups. Based on Tukey’s HSD tests, both current and to-be grandparents have higher levels of happiness than grandparents not-to-be (*p* < 0.001). There were no significant differences between current grandparents and grandparents-to-be. There were significant differences in resilience level (*p* = 0.003) between groups. Specifically, grandparents-to-be reported significantly higher levels of resilience than current grandparents (*p* = 0.003) and grandparents not-to-be (*p* = 0.027). There were significant differences in the three groups’ self-reported physical health (*p* = 0.002). Specifically, grandparents-to-be reported significantly higher levels of resilience than current grandparents (*p* = 0.022) and grandparents not-to-be (*p* = 0.002). There were significant differences in the three groups’ self-reported mental health (*p* = 0.012). Specifically, grandparents-to-be reported significantly higher levels of mental health than current grandparents (*p* = 0.012) and grandparents not-to-be (*p* = 0.041).

**Table 2:** Health and wellbeing of the participants by grandparent status

	Overall (*N* = 1208)	Current (*n* = 507)	To-be (*n* = 275)	Not-to-be (*n* = 426)	F-value
Resilience	7.15 ± 1.62	7.04 ± 1.68	7.44 ± 1.51	7.12 ± 1.60	19.5**
Happiness	20.23 ± 4.58	21.00 ± 4.57	20.44 ± 4.28	19.18 ± 4.61	5.7***
Self-rated physical health	3.41 ± 0.87	3.39 ± 0.90	3.56 ± 0.83	3.33 ± 0.86	6.106**
Self-rated mental health	3.60 ± 0.86	3.55 ± 0.89	3.74 ± 0.79	3.58 ± 0.86	4.433*

Note: ^*^p < 0.05; ^**^p < 0.01; ^***^p < 0.001

### Influence of grandparent status on health and wellbeing

Results of the regression analysis are illustrated in Table 3. Controlling for sex, age, education, financial status, marriage, physical activity, current and grandparents-to-be both reported higher levels of health and well-being than those who do not expect to be grandparents. Specifically, being a current grandparent was significantly related to a 1.429 unit increase in happiness, compared to grandparents not-to-be (*Beta* = 1.429, 95% *confidence interval* = [0.851, 2.006], *p* < 0.001). Being a future grandparent was significantly related to a 0.891 unit increase in happiness, compared to grandparents not-to-be (*Unstandardized coefficients* = 0.891, 95% confidence interval = [0.243, 1.540], *p* < 0.01). Being a future grandparent was significantly related to 0.263 unit increase in resilience compared to grandparents not-to-be (*Unstandardized coefficients* = 0.263, 95% confidence interval = [0.013, 0.512], *p* < 0.05). Being a future grandparent was significantly related to 0.138 unit increase in self-rated physical health compared to grandparents not-to-be (*Unstandardized coefficients* = 0.138, 95% confidence interval = [0.014, 0.262], *p* < 0.05). There was no significant relationship between grandparenting status and self-rated mental health. In addition, grandparenting status did not contribute significant proportions to the variance of self-rated physical and mental health. Even though the values of unstandardized coefficients indicated small to moderate effect sizes, grandparenting status added 12.76 and 8.7% of variance to resilience and happiness, respectively.

**Table 3:** Regression results on predictors of health and wellbeing variables (*N* = 1208)

	Self-rated physical health B[95% CI] (SE)		Self-rated mental health B[95% CI] (SE)		Resilience B[95% CI] (SE)		Happiness B[95% CI] (SE)	
	(1)	(2)	(3)	(4)	(5)	(6)	(7)	(8)
Male (ref: female)	0.174^***^ [0.082, 0.267] (0.047)	0.164^***^ [0.071, 0.258] (0.048)	0.156^***^ [0.065, 0.247] (0.046)	0.145^**^ [0.053, 0.237] (0.047)	0.139 [− 0.047, 0.325] (0.095)	0.099 [− 0.088, 0.287] (0.096)	− 0.374 [− 0.861, 0.113] (0.249)	−0.300 [− 0.787, 0.188] (0.249)
Age	− 0.002 [− 0.010, 0.006] (0.004)	− 0.001 [− 0.009, 0.008] (0.004)	−0.003 [− 0.011, 0.005] (0.004)	−0.001 [− 0.009, 0.008] (0.004)	0.011 [− 0.005, 0.027] (0.008)	0.018^*^ [0.001, 0.035] (0.009)	0.060^**^ [0.018, 0.103] (0.022)	0.032 [− 0.012, 0.077] (0.023)
> Middle school (ref: < middle school)	0.110 [− 0.026, 0.247] (0.069)	0.111 [− 0.026, 0.249] (0.070)	0.195^**^ [0.061, 0.328] (0.068)	0.180^**^ [0.044, 0.315] (0.069)	0.384^**^ [0.111, 0.658] (0.140)	0.346^*^ [0.070, 0.623] (0.141)	−0.191 [− 0.907, 0.525] (0.365)	0.084 [− 0.635, 0.803] (0.367)
Middle school (ref: < middle school)	0.061 [− 0.051, 0.173] (0.057)	0.056 [− 0.057, 0.169] (0.058)	0.087 [− 0.024, 0.197] (0.056)	0.076 [− 0.036, 0.187] (0.057)	0.263^*^ [0.037, 0.490] (0.115)	0.229^*^ [0.002, 0.456] (0.116)	− 0.027 [− 0.618, 0.564] (0.302)	0.112 [− 0.478, 0.703] (0.301)
Finance	0.297^***^ [0.234, 0.359] (0.032)	0.294^***^ [0.232, 0.357] (0.032)	0.296^***^ [0.234, 0.357] (0.031)	0.298^***^ [0.236, 0.360] (0.031)	0.214^***^ [0.088, 0.340] (0.064)	0.216^***^ [0.090, 0.342] (0.064)	1.870^***^ [1.541, 2.199] (0.168)	1.806^***^ [1.478, 2.133] (0.167)
Physical activity	0.290^***^ [0.237, 0.343] (0.027)	0.289^***^ [0.236, 0.343] (0.027)	0.244^***^ [0.192, 0.296] (0.027)	0.245^***^ [0.193, 0.298] (0.027)	0.248^***^ [0.141, 0.355] (0.055)	0.252^***^ [0.145, 0.358] (0.054)	1.070^***^ [0.790, 1.350] (0.143)	1.039^***^ [0.761, 1.316] (0.142)
Married (ref: non-married)	0.089 [−0.032,0.211] (0.062)	0.062 [−0.065,0.188] (0.064)	0.187^**^ [0.067,0.306] (0.061)	0.192^**^ [0.067,0.316] (0.063)	0.073 [−0.172,0.317] (0.125)	0.054 [−0.199,0.308] (0.129)	0.800^*^ [0.161,1.439] (0.326)	0.385 [−0.274,1.044] (0.336)
Current grandparents (ref: not-to-be)		0.029 [−0.082, 0.140] (0.056)		−0.062 [− 0.171, 0.047] (0.056)		− 0.128 [− 0.350, 0.094] (0.113)		1.429^***^ [0.851, 2.006] (0.295)
Grandparents-to-be (ref: not-to-be)		0.138^*^ [0.014, 0.262] (0.063)		0.041 [−0.082, 0.163] (0.062)		0.263^*^ [0.013, 0.512] (0.127)		0.891^**^ [0.243, 1.540] (0.331)
Constant	1.469^***^ (0.313)	1.367^***^ (0.322)	1.883^***^ (0.308)	1.752^***^ (0.317)	4.613^***^ (0.629)	4.160^***^ (0.646)	6.927^***^ (1.646)	8.045^***^ (1.681)
Adjusted R^2^	0.186	0.188	0.184	0.185	0.041	0.047^**^	0.157	0.172^***^

Note: *B* unstandardized cofficients, *CI* confidence interval, *SE* standard error; ^*^p < 0.05; ^**^p < 0.01; ^***^p < 0.001

### Potential moderating role of demographic characteristics

We tested the potential moderating role of five demographic factors: age, sex, marital status, education and financial status. As shown in Table 4, age moderated the relationship between grandparent status and resilience. Specifically, the differences between grandparent-not-to-be and current grandparents in resilience narrowed when age increased (*Unstandardized coefficients* = − 0.040^*^, *standard error* = 0.019). Education and marital status were also significant moderators. Specifically, compared with grandparents-not-be, the advantage in resilience among grandparents-to-be enlarged if the participant has a high educational level (*Unstandardized coefficients* = 0.873^*^, *standard error* = 0.373). The advantage in self-rated mental health among grandparents-to-be were larger if they were not married (*Unstandardized coefficients* = 0.519^*^, standard error = 0.244).

**Table 4:** Significant moderators in the regression models

	Resilience (age as a moderator)	Resilience (education as a moderator)	Mental health (marriage as a moderator)
Male (ref: female)	0.099 (0.096)	0.085 (0.096)	0.145^**^ (0.047)
Age	0.040^**^ (0.015)	0.019^*^ (0.009)	−0.001 (0.004)
> Middle school	0.353^*^ (0.141)	−0.072 (0.226)	0.179^**^ (0.069)
Middle school	0.222 (0.116)	−0.050 (0.209)	0.076 (0.057)
Finance	0.220^***^(0.064)	0.222^***^(0.064)	0.297^***^ (0.032)
Physical activity	0.248^***^(0.054)	0.250^***^(0.054)	0.244^***^ (0.027)
Married (ref: non-married)	0.043 (0.129)	0.058 (0.129)	0.233^**^ (0.082)
Current grandparents (ref: grandparent not-to-be)	2.534^*^ (1.282)	−0.504^*^(0.217)	−0.069(0.060)
Grandparents-to-be (ref: grandparent not-to-be)	1.398(1.543)	−0.101(0.300)	0.007(0.066)
Age* current grandparents	−0.040^*^(0.019)		
Age* grandparents-to-be	−0.017(0.024)		
> Middle school*current grandparents		0.431(0.302)	
Middle school*current grandparents		0.486(0.260)	
> Middle school*grandparents-to-be		0.873^*^(0.373)	
Middle school*grandparents-to-be		0.215(0.343)	
Married* current grandparents			−0.010(0.134)
Married*grandparents-to-be			−0.519^*^(0.244)
Constant	2.691^**^(1.023)	4.375^***^(0.657)	1.799^***^(0.321)
Observations	1208	1208	1208
R^2^	0.057	0.063	0.194
Adjusted R^2^	0.049	0.053	0.187

Note: *B* unstandardized coefficients, *CI* confidence interval, *SE* standard error;

^*^p < 0.05; ^**^p < 0.01; ^***^p < 0.001; only regression models with significant moderators are shown in this table

## Discussion

The present study expands knowledge about the relationship between being grandparents and older people’s health and well-being in Hong Kong. The findings from this study showed that those who did not expect to be grandparents generally reported the lowest level of health and well-being. After controlling other factors, compared with grandparents-not-to-be, current grandparents are more likely to report a higher level of happiness, and grandparents-to-be are more likely to report better self-rated physical health, resilience, and happiness. These findings echo previous research on the relationships between grandparenting and health and wellbeing. For example, using national survey data from the United Kingdom, research has shown that having grandchildren can be beneficial to one’s life satisfaction [40]. A study using data from 11 European countries also reported that entering grandparenthood is associated with increased quality of life and life satisfaction [41]. A recent American study of older Chinese adults noted that feelings of closeness to one’s next generation are positively linked with subjective well-being [19].

Several mechanisms have been cited to rationalize the benefits of grandparenthood in enhancing older people’s well-being. For instance, being a grandparent could be interpreted as a sign of generativity of older people [42]. Interaction with grandchildren is an important source of interpersonal satisfaction and positive affection for older people. Entering into grandparenthood is also associated with a sense of continuity and immortality [40, 41]. A previous study of Hong Kong Chinese older people found, based on social emotional selectivity theory, that grandparenthood can provide interpersonal warmth to older people, especially who perceive limited future time [9].

Findings from the moderating analyses suggested that the differences between three groups of older adults in health and well-being may vary across different subgroups. Specifically, expecting to be a grandparent in the future was found to be compensatory for those non-married or widowed. This may suggest that grandparent-grandchild relationship may compensate for the loss of a spouse and serve an alternative source of emotional support. On the other hand, the difference between current and future grandparents and grandparent-not-to-be in resilience was larger among younger and more highly-educated older adults. This may because older and less educated grandparents may face more stress in grandparenting that may offset the benefits of being a grandparent [13]. In the meantime, according to a previous study in Germany, older adults may perceive less important of becoming a grandparent as their age increases [43]. Therefore, the status of not going to be a grandparent may not have a significant association with their health and well-being.

While extending understandings of the effects of grandparenthood on health and wellbeing, this study carries some limitations. First, the study is a cross-sectional study so no causal relationships can be drawn between. The positive relationship between being a current or future grandparent and health and well-being cannot be conclusively interpreted as the benefits of being a grandparent. More robust studies are needed so that a causal relationship can be claimed. However, the possibility of reverse causality may be limited due to the grandparent status was perceived by older people based on their adult children’s life planning, which may not directly be influenced by their own health and well-being. Nonetheless, multi-wave longitudinal studies and experimental designs should be adopted in the future to further investigate these relationships. Second, the findings from this study cannot be generalized to other Chinese population. For instance, the contexts around grandparenting and the role of grandparents are quite different in Mainland China than in Hong Kong due to the socio-economic dynamics. Third, the study adopted a limited number of controlling variables so that there may be other unobservable variables that simultaneously influence grandparenthood and health outcomes variables. Factors such as the participants’ own perception and attitudes of grandparenting may influence the link between grandparent status and older adults’ health and well-being. Other interpersonal relationship (e.g., with adult children, spouse, and friends) were not controlled in the study. Future studies should include more controlling variables in more domains. Moreover, future studies should also address societal factors that may influence older adults’ appraisal of being a grandparent. For instance, the social unrest in Hong Kong since the second half of 2019 has posed challenges to intergenerational relationship both within the family and in the society due to the differentiated political attitudes among the younger and older adults. Multi-level studies should be conducted in the future to better understand the interaction between societal, familial, and individual factors. Fourth, the current study only focused on the status of being a grandparent or not, but did not capture the details of grandparenting, such as living arrangement, types and amount of grandparenting activities. For those current grandparents, these characteristics may influence their health and well-being.

Despite these imitations, this study offers some implications for future research, policy-making and elderly services planning. First, the World Health Organization’s Healthy Ageing Framework has identified health services, long term care, and environments as crucial domains for supporting healthy aging [44]. The development of both physical and social environments to promote capacity-enhancing behaviours is essential to sustain ageing people. Intergenerational relationship, namely grandchild-grandparent relationship provides an excellent entry point for social and community health intervention for grandparents to build and enhance capacity. To promote intergenerational connection is helpful for older adults to gain social and emotional support, especially for those who are in lack of other types of interpersonal relationship. Even though the effect sizes computed in this study were only small to medium, they may still indicate significant real world differences. On the one hand, effect sizes and *p*-value should be both considered while interpreting the associations between two variables. Findings from this study are consistent with previous studies that suggested a significant relationship between grandparenthood and older people’s health and well-being [40, 41]. On the other hand, previous studies have shown that subjective well-being, namely happiness, mostly contributed to pre-exiting variables such as personality and genetic factors, tend to be stable in late adulthood [45, 46]. Therefore, even a small effect size may still indicate unignorable group differences that warrant future investigation. In addition, our study has also found that the positive relationship between grandparenting and health are not universal across subgroups. Therefore, training programs and interventions should be implemented to increase the grandparenting skills of older adults, especially those disadvantaged to relieve the potential stress and conflict related to grandparenting. Further detailed examination of the specific characteristics of different types of grandparents or grandparents-to-be would be helpful to identify what can be done to further support these older people in their later life.

On the other hand, older people are less likely to be a grandparent or have as many as grandchildren nowadays than previous times. The decreasing fertility rate, women’s increasing levels of education, rising individualism, and high costs of child rearing contributed to delayed marriage and childbirth [47–51]. The lower probability of the birth of grandchildren may prevent older people from enjoying the benefits of grandparenthood, especially in cultural contexts where social expectations towards childbirth, parenthood, and grandparenthood are still prevalent [52]. As older people who are not going to be grandparents may miss one of the sources of interpersonal support, policies and practices should aim to identify and address their specific vulnerabilities. Other types of active aging programs and interventions should be developed to compensate for the potential challenges associated with interpersonal relationships and sense of loss due to not being a grandparent. For instance, more opportunities in developing other types of interpersonal relationship, such as neighbours, friends, or non-familial intergenerational programs should be introduced. Studies from multiple countries have proved the effectiveness of intergenerational programs outside the familial settings in promoting older adults’ health and well-being [53]. The comparison between grandparent-grandchild relationship and other types of interpersonal relationship can be further conducted to understand the advantages and disadvantages of different interpersonal activities. Moreover, attention should be paid to social norms, with efforts to challenge and minimize stigmatization associated with not having grandchildren, in order to contribute to supportive sociocultural environments for different groups of older people.

## Conclusions

The present study provides preliminary evidence regarding the positive relationship between grandparenthood and older Hong Kong Chinese people’s health and well-being. Being a grandparent remains important for older people in Chinese culture, but it may not necessarily bring benefits for all subgroups of older people. This study calls for more future studies to understand the health outcomes of grandparenting or non-grandparenting.

## Supplementary Information

**Additional file 1:.** Questionnaire used for the study.

## Data Availability

The datasets used and/or analysed during the current study are available from the corresponding author on reasonable request.
