# Supplementary material for: To be or not to be: relationship between grandparent status and health and wellbeing
Source: BMC Geriatr. 2021 Mar 24;21:204. doi: 10.1186/s12877-021-02052-w (PMC7989105; doi:10.1186/s12877-021-02052-w)
Supplement: Supplementary file 1 — Additional file 1:. Questionnaire used for the study. [file 12877_2021_2052_MOESM1_ESM.pdf]

Part One: Retirement Experience and Expectation

Screening question 1:

1. Have you retired?

1) Yes 2) No

- If yes, 1a: How long have you been retired? 1. Less than one year 2=one to three years 3= above three years

1) Less than one year 2) one to three years 3) above three years

- If no, 1b: when would you expect to retire?
  In _______ years

- Are you currently engaging in paid work?

1) No 2) Half-time job/ Part-time job 3) Full-time job

2a. [For retirees] Overall speaking, are you satisfied about life during retirement?

Very unsatisfied				Very satisfied
1	2	3	4	5

2b. [For future retirees] How would you expect your satisfaction level in retirement?

Very unsatisfied				Very satisfied
1	2	3	4	5

3. [For retirees] Before you retired, had you done the following things to prepare for your retirement?
[For future retirees] Have you made the following preparations for your retirement?

	1 = yes	0 = no
Financial Planning for Retirement:		
a) Saving for retirement.		

	1 = yes	0 = no
b) Bought flat or apartment for retirement.		
c) Bought stocks, funds, or bonds for long-term investment for retirement.		
d) Bought life or saving insurance policy.		
e) Bought accident insurance policy.		
Health Planning for Retirement:		
f) Bought medical insurance policy.		
g) Exercise regularly (at least twice a week).		
h) Arrange medical checkup periodically (at least once every 2 years).		
i) Quit or start to quit habits that are hazardous to health.		
Living Arrangement Planning for Retirement:		
j) Cultivate hobbies to keep busy during retirement.		
k) Plan for retirement living arrangement.		
l) Plan for retirement daily life activities.		
Psychological Planning for Retirement:		
m) Participate in workshops, seminars, or courses on retirement.		
n) Read books or articles about retirement.		
o) Watch television or listen to radio programs concerning retirement.		

	1 = yes	0 = no
p) Discuss with immediate family members about your retirement.		
If yes, which immediate family members have you discussed with? (can choose multiple answers): 1. Spouse 2. Children. 3. Siblings. 4. Parents		
q) Discuss with relatives, friends, and colleagues about your retirement.		
r) Discuss retirement with retired people.		
s) Discuss retirement with social services workers.		

Part Two: Grandparenting Experience and Expectations

Section 1: While some people are or would expect themselves to be a grandparent one day and some may not expect this to happen, we would like to explore your opinions about being a grandparent.
To what degree do you agree with the following the statements? (1=strongly disagree, 5=strongly agree)

a) Grandparents are obligated to take care of their own grandchildren	1	2	3	4	5
b) Grandparenting helps older adults maintain a positive life	1	2	3	4	5
c) Grandparenting can improve life satisfaction of the older adults.	1	2	3	4	5
d) Grandparenting is much more important than other leisure activities for retirees.	1	2	3	4	5
e) Grandparents are responsible for assisting their own grandchildren. (i.e. when parents are divorced or in illness)	1	2	3	4	5
f) Grandparents are responsible for offering their own grandchildren and their families financial support.	1	2	3	4	5
g) Grandparents have the duty of assisting children to take care of young grandchildren	1	2	3	4	5
h) Grandparents have the duty to help children in guiding grandchildren.	1	2	3	4	5

Screening question 2:

- Do you currently have grandchildren?
  1) Yes (to Section 2)   0) No

- If not currently having grandchildren, do you expect to have grandchildren in the future?
  1) Yes (to Section 3)   0) No

- Would you like to have grandchildren in the future?
  1) Yes (to Section 3)   2) No (to **Part three**)

Section 2: **[for current grandparents only]**

1. How many grandchildren do you have? ____ (if >1, choose the one with whom you get along with most frequently)

2. What is his/her age group?
   1) 0 to 3 years   2) 4-6 years   3) 6-10 years
   4) 10-15 years   5) 16-20 years   6) 21 years and above

3. Does he/she currently live in the same household with you? 1=yes 2=no
   1) Yes   2) No

4. Does his/ her father or the mother live with you?
   1) Yes   2) No

5. How often have you done the following activities with your grandchild in the past year? Ideally, how often would you like to do the following activities?

	How often have you done the following activities with your grandchild in the past year?					Ideally, how often would you like to do the following activities?		
	Never	Once in several month s	Once per month	Once or several times per week	Daily	Less frequent than current	Same as current	More frequen tly than current
See him/her face to face								
Communicate with him/ her face to face								
See him/her over the phone/ the Internet								
Chat with him/her online or by phone								
Send him/her a gift (including occasional pocket money)								
Help with his/her daily routines (e.g. clothing, food, housing and transport)								
Share life experience (e.g. attitude in dealing with matters and persons, politeness)								

	How often have you done the following activities with your grandchild in the past year?					Ideally, how often would you like to do the following activities?		
	Never	Once in several month s	Once per month	Once or several times per week	Daily	Less frequent than current	Same as current	More frequen tly than current
Teach him/ her life skills (e.g. taking care of himself/ herself, doing household chores, time management, financial management								
Do indoor activity with him/ her solely (or with your spouse) (e.g. playing chess at home, playing mahjong , visiting museums, participating in cultural and arts activities)								
Do outdoor activity with him/ her solely (or with your spouse) (e.g. hiking, visiting theme park)								

	How often have you done the following activities with your grandchild in the past year?					Ideally, how often would you like to do the following activities?		
	Never	Once in several month s	Once per month	Once or several times per week	Daily	Less frequent than current	Same as current	More frequen tly than current
Do indoor activity with him/ her and other family members (e.g. playing chess at home, playing mahjong , visiting museums, participating in cultural and arts activities)								
Do outdoor activity with him/ her and other family members (e.g. hiking, visiting theme park)								

Screening Question 3: Is your grandchild currently attending school?

1) Yes （answer the following question）
2) No（to Question 6）

	How often have you done the following activities with your grandchild in the past year?					Ideally, how often would you like to do the following activities?		
	Never	Once in several months	Once per month	Once or several times per week	Daily	Less frequent than current	Same as current	More frequently than current
Help with his/ her studies (e.g. solving homework problems, doing revision)								
Deal with his/ her school-related issues (e.g. attending parents' day, reading and signing notice, attending open day)								

6. How close are you with your grandchild?

Not close at all									Very close
1	2	3	4	5	6	7	8	9	10

7. Taken everything together, how would you describe with your elation with your grandchild?

Not close at all									Very close

1	2	3	4	5	6	7	8	9	10

8. How close do you think is your relationship with the parents of your grandchild?

Not close at all									Very close
1	2	3	4	5	6	7	8	9	10

9. Generally speaking, how would you describe with your relationship with the parents of your grandchild?

Not close at all									Very close
1	2	3	4	5	6	7	8	9	10

10. As a grandparent, to what extent have you encountered difficulties in planning or carrying out the following activities? (1=Not difficult at all, 5=Very difficult)

Communicate with your adult children and in-laws	1	2	3	4	5
Communicate with your grandchildren	1	2	3	4	5
Come up with activities you think your grandchildren will enjoy					
Play with your grandchildren solely (or with your spouse)	1	2	3	4	5
Play with your children and grandchildren	1	2	3	4	5
Help with your grandchildren's daily routines (e.g. clothing, food, accommodation and mobilization)	1	2	3	4	5
Share life experience with your grandchildren (e.g. attitude in dealing with matters and persons, politeness)	1	2	3	4	5
Teach your grandchildren's life skills (e.g. taking care of himself/ herself, doing household chores, time management, financial management	1	2	3	4	5
Teach and discipline grandchildren	1	2	3	4	5
**(For participant whose grandchild is attending school)** Help with your grandchildren's study (e.g. solving homework problems, doing revision)	1	2	3	4	5
**(For participant whose grandchild is attending school)** Handle other school-related issues of your grandchildren (e.g.	1	2	3	4	5

attending Parents' Day, reading and signing bulletin, attending open day)					

11. Which of the following reasons lead to the above difficulties you encountered?
    1. You are lacking relevant knowledge and skills
    2. Your health status is not well
    3. Your financial status is not well
    4. Your availability does not accommodate grandchildren's
    5. You live far away from your grandchildren
    6. There is a generation gap between you and your grandchildren
    7. Your views on how to discipline grandchildren are different from your children's
    8. Your views on how to take care of your grandchildren are different from your children's
    9. Others (please specify): _________

12. Would you be interested in training/learning how to improve your skills in the following areas? (1=Not interested at all, 5=Very interested)

Ways to adapt to changes after being grandparent	1	2	3	4	5
Communication skills with your adult children and in-laws	1	2	3	4	5
Communication skills with your grandchildren	1	2	3	4	5
Skills on taking care of your grandchildren	1	2	3	4	5
Skills on disciplining your grandchildren	1	2	3	4	5
Knowledge on child psychology	1	2	3	4	5
Primary and secondary school knowledge	1	2	3	4	5
Skills on handling conflicts	1	2	3	4	5
Skills on planning activities	1	2	3	4	5
Presentation skills	1	2	3	4	5
Skills on using information technology	1	2	3	4	5

Section 3: **[for who expect/ want to be grandparents only]**

1. If you are a grandparent, how often in a year "would you like" to do the following activities with your grandchild?

	Never	Once in several months	Once per month	Once or several times per week	Daily
See him/her face to face					
Communicate with him/ her face to face					
Send him/her a gift (including occasional pocket money)					
Help with his/her daily routines (e.g. clothing, food, housing and transport)					
Share life experience (e.g. attitude in dealing with matters and persons, politeness)					
Teach him/ her life skills (e.g. taking care of himself/ herself, doing household chores, time management, financial management					
Do indoor activity with him/ her solely (or with your spouse) (e.g. playing chess at home, playing mahjong, visiting museums, participating in cultural and arts activities)					
Do outdoor activity with him/ her solely (or with your spouse) (e.g. hiking, visiting theme park)					

	Never	Once in several months	Once per month	Once or several times per week	Daily
Do indoor activity with him/ her and other family members (e.g. playing chess at home, playing mahjong , visiting museums, participating in cultural and arts activities)					
Do outdoor activity with him/ her and other family members (e.g. hiking, visiting theme park)					
Help with his/ her study (e.g. solving homework problems, doing revision)					
Handle his/ her school issues (e.g. attending parents' day, reading and signing bulletin, attending open day)					

2. Do you have any preparation or planning for grandparenting?
   1) Yes   0) No (to **Part 3**)

3. Through what ways do you prepare to be a grandparent? Choose all apply:
   1) Attending classes
   2) Reading books or online materials
   3) Participating in intergenerational volunteering activities
   4) Others (please specify): ______

4. How close do you think your relationship is with the parents of your grandchild?

Not close at all									Very close
1	2	3	4	5	6	7	8	9	10

5. Taken everything together, how would you describe your relationship with the parents of your grandchild.

Not close at all									Very close
1	2	3	4	5	6	7	8	9	10

6. If you are grandparent, to what extent will you encounter difficulties in planning or carrying out the following activities? (1=Not difficult at all, 5=Very difficult)

Communicate with your adult children and in-laws	1	2	3	4	5
Communicate with your grandchildren	1	2	3	4	5
Come up with activities you think your grandchildren will enjoy	1	2	3	4	5
Play with your grandchildren solely (or with your spouse)	1	2	3	4	5
Play with your children and grandchildren	1	2	3	4	5
Help with your grandchildren's daily routines (e.g. clothing, food, housing and transport)	1	2	3	4	5
Share life experience with your grandchildren (e.g. attitude in dealing with matters and persons, politeness)	1	2	3	4	5
Teach your grandchildren's life skills (e.g. taking care of himself/ herself, doing household chores, time management, financial management	1	2	3	4	5
Teach and discipline grandchildren	1	2	3	4	5
Help with your grandchildren's study (e.g. solving homework problems, doing revision)	1	2	3	4	5
Deal with the matters related to your grandchildren's study (e.g. attending parents' day, reading and signing notice, attending open day)	1	2	3	4	5

7. Which of the following reasons lead to the above difficulties you expect to encounter?
    1. You are lacking relevant knowledge and skills
    2. Your health status is not well
    3. Your financial status is not well
    4. Your availability does not accommodate grandchildren's
    5. You live far away from your grandchildren
    6. There is a generation gap between you and your grandchildren
    7. Your views on how to discipline grandchildren are different from your children's
    8. Your views on how to take care of your grandchildren are different from your children's
    9. Others (please specify): ________

8. Would you be interested in learning to improve your skills in the following aspects? (1= Not interested at all, 5= very interested)

Ways to adapt to changes after being grandparent	1	2	3	4	5
Communication skills with your adult children and in-laws	1	2	3	4	5
Communication skills with your grandchildren	1	2	3	4	5
Skills on taking care of your grandchildren	1	2	3	4	5
Skills on disciplining your grandchildren	1	2	3	4	5
Knowledge on child psychology	1	2	3	4	5
Primary and secondary school knowledge	1	2	3	4	5
Skills on handling conflicts	1	2	3	4	5
Skills on planning activities	1	2	3	4	5
Presentation skills	1	2	3	4	5
Skills on using information technology	1	2	3	4	5

Part Three: Health and Wellbeing

1. In general, how do you rate your physical health status?
   1) Very poor 2) Poor 3) Fair 4) Good 5) Very good

2. In general, how do you rate your mental health status?
   1) Very poor 2) Poor 3) Fair 4) Good 5) Very good

3. In general, I consider myself:

not a very happy person						a very happy person
1	2	3	4	5	6	7

4. Compared to most of my peers, I consider myself:

less happy						more happy
1	2	3	4	5	6	7

5. Some people are generally very happy. They enjoy life regardless of what is going on, getting the most out of everything. To what extent does this characterization describe you?

not at all						a great deal
1	2	3	4	5	6	7

6. Some people are generally not very happy. Although they are not depressed, they never seem as happy as they might be. To what extend does this characterization describe you?

not at all						a great deal
1	2	3	4	5	6	7

7. Including walking, doing housework, hobbies, and other activities, how active will you rate yourself on doing physical activities?
   1) Very low 2) Low 3) Fair 4) High 5) Very high

8. Please indicate the extent of your agreement with the two following statements in the . If the specific situation has not happen, please indicate how you think you would have reacted if it really happen.

	0= Never	1	2	3	4=Almost all the time
Able to adapt to change					
Tend to bounce back after illness or hardship					

9. Are you married? :

1) Yes (To Question 11)　　2) No

10. If you are not married, do you have a long-time partner?

1) Yes (To Question 12)　　2) No (To Part Four)

11. Based on your feelings about your current marital relationship, please choose the answer that can represent your feelings the most. (1=extremely dissatisfied, 7=extremely satisfied)

How satisfied are you with your marriage?	1	2	3	4	5	6	7
How satisfied are you with your husband/wife as a spouse?	1	2	3	4	5	6	7
How satisfied are you with your relationship with your couple relationship?	1	2	3	4	5	6	7

12. Based on your feelings about your current partnership, please choose the answer that can represent your feelings the most. (1=extremely dissatisfied, 7=extremely satisfied)

How satisfied are you with your partnership?	1	2	3	4	5	6	7
How satisfied are you with your partner?	1	2	3	4	5	6	7
How satisfied are you with your relationship with your partner?	1	2	3	4	5	6	7

Part Four: Demographic Information

1. What is your gender?
   1) Male   2) Female

2. What is your current age?
   _______ years old

3. What is your highest educational attainment level?
   1) No formal education
   2) Primary school (never graduated)
   3) Primary school (graduated)
   4) Middle and high school (never graduated)
   5) Middle and high school
   6) Non-degree diploma/ Certificate
   7) College
   8) Postgraduate and above

4. In general, what is your current financial status?
   1) Very poor   2) Poor   3) Fair   4) Good   5) Very good
